# Leukemia Inhibitory Factor Induces Proopiomelanocortin via CRH/CRHR Pathway in Mouse Trophoblast

**DOI:** 10.3389/fcell.2021.618947

**Published:** 2021-07-19

**Authors:** He Wang, Hiromi Sakata-Haga, Hiroko Masuta, Mitsuhiro Tomosugi, Tsuyoshi Tsukada, Hiroki Shimada, Daisuke Sakai, Hiroki Shoji, Toshihisa Hatta

**Affiliations:** ^1^ Department of Anatomy, Kanazawa Medical University, Uchinada, Japan; ^2^ Department of Obstetrics, The First Hospital of China Medical University, Shenyang, China; ^3^ Department of Medical Science, Kanazawa Medical University, Uchinada, Japan; ^4^ Department of Biology, Kanazawa Medical University, Uchinada, Japan

**Keywords:** leukemia inhibitory factor, corticotropin-releasing hormone, corticotropin-releasing hormone receptor, proopiomelanocortin, mouse trophoblast stem cells, mouse placenta

## Abstract

We previously showed that maternal leukemia inhibitory factor (LIF) induces placental production of adrenocorticotropic hormone (ACTH), which stimulates fetal nucleated red blood cells to further secrete LIF and promote neurogenesis in rodent brains. However, the underlying mechanism of LIF-dependent ACTH induction remains unclear. Recently, we found that LIF induces corticotropin-releasing hormone (CRH) in mouse trophoblast stem cells. This finding supports the results of a previous study that CRH, which is produced by the placenta, induces placental ACTH production. In this study, we examined whether the effects of LIF are mediated by the induction of *Pomc* via CRH upregulation in mouse trophoblast. *In vivo*, protein levels of LIF and CRH peak in mouse placenta at 13.5 days post coitum. In mouse placenta, *Crh* mRNA and protein levels significantly increased 3 h after intraperitoneal injection of LIF (5 μg/kg body weight) into dams at 13.5 days post coitum. We also examined the effect of LIF-induced CRH on the expression of *Pomc* induced by LIF in mouse trophoblast stem cells *in vitro*. After LIF supplementation for 3 days, we found that the increased expression of *Crh*-induced by new supplementation of LIF was earlier than that of *Pomc*. Furthermore, LIF-induced upregulation of *Pomc* in mouse trophoblast stem cells was attenuated by inhibition of the CRH/CRHR1 pathway, whereas LIF-induced secretion of ACTH was attenuated by inhibition of the JAK/STAT3 pathway. Therefore, LIF indirectly increases placental *Pomc* expression through the CRH/CRHR1 pathway, and placental ACTH secretion is induced directly by LIF via the JAK/STAT3 pathway.

## Introduction

The placenta is essential to mammalian pregnancy, with many roles that go beyond fetal nutrition, including both endocrine and immune functions (Coe and Lubach, 2014). As pregnancy progresses, the placenta becomes an important organ for physiological interactions between the mother and embryo (Sandman, 2016). The placenta expresses and secretes a variety of hormones, cytokines, and receptors by activating different maternal-fetal pathway signals to make maternal pregnancy tolerable, promote fetal development (Johnson, 2011), and, ultimately, ensure good pregnancy outcome.

Leukemia inhibitory factor (LIF) is a pleiotropic cytokine essential for development of the hematopoietic and central nervous systems. Previous research abundantly supports the fact that LIF is a critical player in placental development and establishment of a healthy pregnancy (Winship et al., 2015). It is well known that LIF is expressed in both adult and fetal pituitary cells, and plays an important role in the differentiation and development of neurons. Our previous study on rodents showed that maternal LIF induces proopiomelanocortin (POMC) expression and placental secretion of adrenocorticotropic hormone (ACTH), which stimulates fetal nucleated red blood cells to release LIF, and consequently, enhance neuron production in the ventricular zone of fetal cerebral neocortex (Simamura et al., 2010, 2015). However, the underlying mechanism by which maternal LIF induces placental ACTH secretion remains unclear. Recently, we found that LIF induces corticotropin-releasing hormone (CRH) in differentiated mouse trophoblast stem cells (mTSCs) (Wang et al., 2020). CRH is a peptide hormone in the hypothalamus, and its main function is stimulation of ACTH synthesis in the pituitary as part of the hypothalamic-pituitary-adrenal axis. In addition, it was reported that placental CRH exerts a local paracrine action on the production of Pomc and secretion of ACTH from the placenta (Petraglia et al., 1987; Margioris et al., 1988). Based on these findings, we hypothesized that this LIF/CRH pathway is involved in LIF-induced expression of placental *Pomc* and secretion of placental ACTH. However, there is evidence that LIF directly acts via the Janus kinase/signal transducer and activator of transcription (JAK/STAT) pathway to potently stimulate *Pomc* transcription and ACTH secretion from pituitary corticotrophs and cells from the murine AT-20 corticotroph cell line (Ray et al., 1996; Chesnokova and Melmed, 2002). In this study, we examined whether LIF indirectly acts via CRH/CRHR to stimulate *Pomc* expression and ACTH secretion from mouse trophoblast *in vitro* and *in vivo*.

## Materials and Methods

### Cell Culture and Treatments

Protocols for mTSC maintenance and differentiation were based on previously published methods (Erlebacher et al., 2004; Natale et al., 2009; Wang et al., 2020). For differentiation on culture day 8, cells were treated with recombinant mouse LIF (Millipore, Darmstadt, Germany) after which the medium was changed daily. LIF was continuously added to the medium of LIF-treated groups until the end of the experiment. We chose six inhibitors specifically, JAK Inhibitor 1 (Merck Life Science, Darmstadt, Germany), LY294002 (Wako, Osaka, Japan), U0126 (Wako), asstressin (Sigma Aldrich, St. Louis, MO), antalarmin (Sigma Aldrich, St. Louis, MO, United States), and asstressin 2B (Sigma Aldrich) to suppress LIF-activated JAK/STAT3, PI3K/AKT, MAPK, CRH/CRHR1 and 2, CRH/CRHR1, and CRH/CRHR2 pathway, respectively. mTSCs were incubated with LIF for 3 days from day 8 to 11. On day 11, mTSCs were incubated with or without the respective inhibitor, JAK Inhibitor 1, LY294002, U0126 (20 μM each), asstressin (1 μM), antalarmin (1 μM), or asstressin 2B (1 μM) for 30 min. After changing to new medium containing LIF, cells were analyzed at 3, 6, and 12 h and cell media were analyzed at 15 min. In addition, mTSCs were harvested for real-time RT-PCR or western blot. ACTH concentration in culture medium was measured by sandwich ELISA, details of which are described in section “Sandwich ELISA” In all experiments, the number of samples in each group was between three to eight.

### Animals and Treatments

Female C57BL/6J mice aged 8–24 weeks were used in this study. The mice were maintained under standard laboratory conditions. Each female mouse was housed with a male mouse overnight; noontime of the day when a vaginal plug was found in the morning was designated as 0.5 day post coitum (dpc). To minimize suffering, dams were anesthetized by intraperitoneal injection of a mixture of medetomidine (0.3 mg/kg body weight [BW]), midazolam (4 mg/kg BW), and butorphanol (5 mg/kg BW). All procedures involved in this study were performed in strict accordance with the guidelines for the Care and Use of Laboratory Animals of Kanazawa Medical University. The protocol was approved by the Ethics Committee on Animal Experiments of the Kanazawa Medical University (Permit Number: 2012–25, 2013–16, 2014–2, 2014–33).

Some of the pregnant mice were not treated. These pregnant mice underwent laparotomy under anesthesia for collection of placental tissues on 13.5, 15.5, and 17.5 dpc.

The other part of pregnant mice, excluding controls, received an intraperitoneal injection of LIF at 1, 5, and 25 μg/kg BW on 13.5 dpc (Simamura et al., 2010; Tsukada et al., 2015). Our controls were pregnant mice treated with saline. These pregnant mice underwent laparotomy under anesthesia for collection of placental tissues 3 h after LIF or saline injection.

Placental tissues were used for immunohistochemistry, quantitative RT-PCR and western blot analyses, details of which are described in sections “Immunohistochemistry”, “Gene expression by quantitative RT-PCR”, and “Western blot”, respectively.

### Immunohistochemistry

Fresh placental tissue was washed twice with saline and fixed with 4% paraformaldehyde. Paraffin placenta sections were cut 10-μm thick for immunofluorescence staining, performed by heat-mediated antigen retrieval using 10-mM citrate buffer (pH 6.0) at 100°C under microwave irradiation for 10 min. Sections were blocked with 1% BSA in phosphate-buffered saline (PBS) containing 0.1% Triton X-100. Sections were incubated overnight at 4°C with a rabbit anti-mouse CRF antibody (2 mg/mL; Bioss; RRID:AB_10885736) and a rabbit anti-mouse LIF receptor (LIFR) antibody (0.2 mg/mL; Santa Cruz Biotechnology) after washing with PBS. Non-immunized rabbit IgG (2 μg/mL, Dako Cytomation) was used as the negative control. Sections were incubated with Alexa Fluor 594 conjugated anti-rabbit IgG (4 mg/mL) and counterstained with Hoechst 33342 (5 mg/mL, Thermo Fisher Scientific) containing RNase A (10 mg/mL) after washing with PBS. Furthermore, sections were then mounted with PermaFluor (Thermo Scientific) and observed with an LSM 710 confocal laser scanning microscope (Zeiss).

### Gene Expression by Quantitative RT-PCR

Total RNA of placenta and mTSCs was extracted using a RNeasy mini kit (Qiagen, Valencia, CA, United States). Expression levels of *Crh* were determined using a gene expression assay (Applied Biosystems, Foster City, CA, United States). cDNA was synthesized using SuperScript III reverse transcriptase (Invitrogen), and quantitative PCR was performed using cDNA with TaqMan gene expression master mix (Takara Bio, Kusatsu, Japan). *Crh* (Mm01293920_s1) expression was normalized to the housekeeping genes of 18S ribosomal RNA (Applied Biosystems) using the 2^–Δ^ ^Δ^ ^*Ct*^ method (Livak and Schmittgen, 2001). *Pomc* RT-PCR was performed using TB Green Premix Ex Taq II (Tli RNaseH Plus, Takara Bio) and a Thermal Cycler Dice Real-Time System III instrument (Takara Bio). Each PCR reaction consisted of 6.25 μL TB Green Premix Ex Taq II, 4.5 μL water, 1 μL cDNA, and 0.5 μL (0.39 μM) *Pomc* (NM_008895) gene-specific primer (Simard et al., 2010). The cycle threshold for *Pomc* amplification was normalized to the expression level of 18S ribosomal RNA. Reactions were performed in triplicate.

### Sandwich ELISA

Adrenocorticotropic hormone concentration in mTSC culture medium was detected using an antimouse ACTH antibody (1:100, Santa Cruz Biotechnology) for capture and a biotin-labeled anti-ACTH antibody (1:5000, Chemicon) for detection. Validation of the ACTH assay (*n* = 3) showed a sensitivity of 0.25 nM, an intra-assay coefficient of variance of 7.21%, and an inter-assay coefficient of variance of 5.74%. An ACTH fragment (ACTH 1–24 of human/rat) was used as a standard. All plates were blocked with buffer (Starting Block, Pierce, Waltham, MA, United States) and biotin was exposed to an ExtrAvidin-peroxidase conjugate (Sigma Aldrich). HRP activity was detected with 1-Step Ultra TMB Substrates (Pierce), and triplicate samples were measured using a 2104 EnVision Multilabel Plate Reader (PerkinElmer, Waltham, MA, United States). All medium samples were diluted 1:10 before proceeding.

### Western Blot

Placental tissues at 13.5, 15.5, and 17.5 dpc were used for western blot analysis. Each placenta was homogenized in 400 μL PRO-PREP protein extraction solution (iNtRON Biotechnology, Kirkland, WA, United States). After homogenization, placental lysate was incubated for 20 min on ice and centrifuged at 13,000 rpm at 4°C for 5 min. The supernatant was collected, and protein content was measured using the Micro BCA protein assay kit (Pierce). Protein samples were denatured in buffer (Pierce) for 5 min at 100°C, separated by SDS-PAGE, and transferred to a polyvinylidene fluoride membrane (Invitrogen). After blocking, membranes were incubated overnight at 4°C with an anti-CRF polyclonal antibody (1:300, Bioss, RRID:AB_10885736) and an anti-LIF polyclonal antibody (1:200, Santa Cruz Biotechnology). β-actin (1:1000, Santa Cruz Biotechnology, RRID:AB_626632) was used as a loading control. Membranes were washed using Tris–buffered saline with 0.05% Tween 20 and incubated at 27°C with horseradish peroxidase-conjugated antirabbit (1:5000, MBL, Nagoya, Japan) or antimouse (1:5000, MBL) secondary antibody. Signals were detected using an Enhanced Chemiluminescence Reagent (Pierce). For reblotting, Restore Plus Western Blot Stripping Buffer (Thermo Fisher Scientific) was used to strip membranes. ImageJ was used to quantify band intensities in every membrane. Each experiment was repeated two or three times.

### Statistical Analysis

All data in this study are expressed as mean ± standard error of the mean (SEM) as presented in figure legends. Data analyses were performed using GraphPad Prism 5.0 (GraphPad Software, La Jolla, CA, United States). For all targets, datasets underwent F test to determine equality of variances. Unpaired two-tailed Student’s *t*-test was performed to compare two experimental groups. Moreover, ANOVA with Tukey *post hoc* test was performed for multiple comparisons. A *P*-value < 0.05 was accepted as statistically significant.

## Results

### CRH and LIF Protein Levels in Mouse Placenta During Development

**FIGURE 1:**
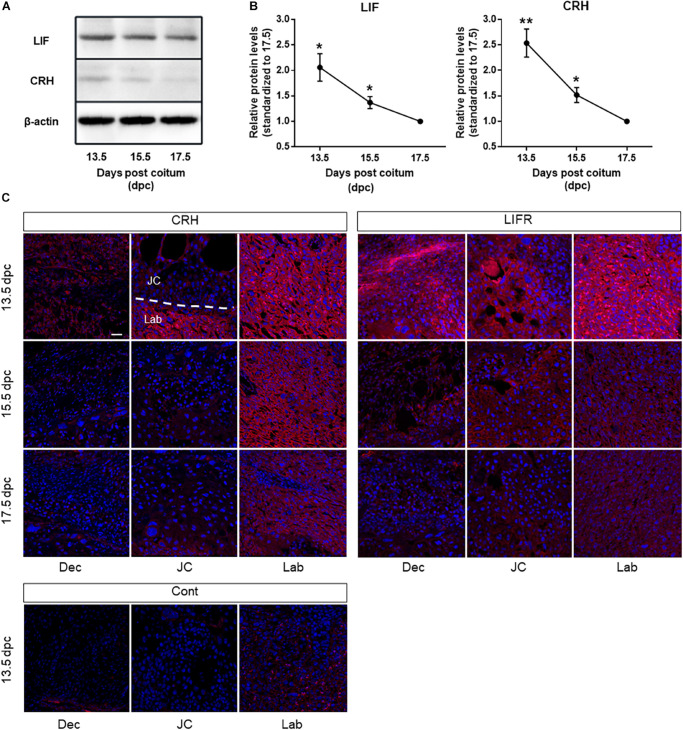
CRH and LIF expression in mouse placenta during development. **(A)** Representative immunoblots and **(B)** quantification of CRH and LIF protein levels in mouse placenta at 13.5, 15.5, and 17.5 dpc. The housekeeping protein β-actin (42 kDa) was used as a loading control. Densitometry reveals a significant increase in CRH peptide and LIF at day 13.5 dpc in mouse placenta (*n* = 4 of each group), with each point standardized to 17.5 dpc. All data are expressed as mean ± SEM. *P < 0.05, **P < 0.01 vs. control (Student’s *t*-test). **(C)** Placental sections obtained from mice at 13.5, 15.5, and 17.5 dpc underwent immunofluorescence staining using a CRH-specific antibody (*left panel*) and LIFR-specific antibody (*right panel*). Nuclei were stained with Hoechst 33342 (*blue*). *Scale bar* 50 μm. *Dec* decidua, *JC* junctional zone, *Lab* labyrinth, *Cont* negative control at13.5 dpc.

Previously, we demonstrated that LIF induces CRH in mTSCs. In this study, we examined CRH and LIF levels in mouse placenta at different days of gestation namely, 13.5, 15.5, and 17.5 dpc. We found that protein levels of CRH and LIF were higher at 13.5 dpc compared with that found at 15.5 and 17.5 dpc (Figures 1A,B), and furthermore, CRH and LIF protein levels decreased following placental development. CRH and LIFR were widely expressed in the decidua, trophoblast giant cells, and syncytiotrophoblast layer of mouse placenta, particularly in the syncytiotrophoblast cells at 13.5 dpc (Figure 1C). However, spongiotrophoblast cells exhibited lower intensity compared with syncytiotrophoblast cells. Immunoreactivity in the syncytiotrophoblast cells decreased to 17.5 dpc (Figure 1C), which was consistent with the CRH peptide level in mouse placenta (Figure 1B). Interestingly, spatial and temporal expression patterns of LIFR in the placenta were also consistent with those of CRH.

### LIF-Induced CRH in Mouse Placenta at 13.5 dpc

**FIGURE 2:**
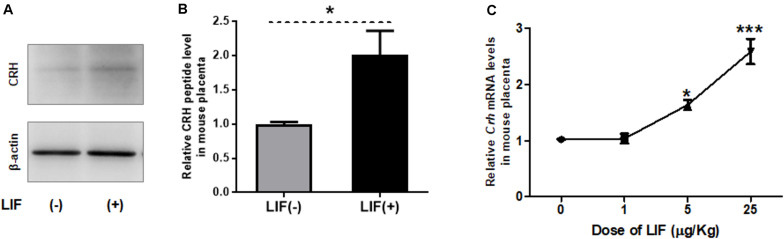
LIF upregulates CRH in mouse placenta. Recombinant LIF is injected intraperitoneally to pregnant mice on 13.5 dpc, followed by placental tissue collection after 3 h. **(A, B)** Using Western blot, placental CRH peptide level is measured after maternal treatment with LIF (5 μg/kg BW). **(A)** Representative immunoblots and **(B)** quantification of CRH peptide level in mouse placenta with (*positive symbol*) or without (*negative symbol*) LIF treatment (*n* = 6). All data are expressed as mean ± SEM. *P < 0.05 vs. the LIF (*negative symbol*) group (Student’s *t*-test). **(C)** Using real-time RT-PCR, *Crh* mRNA levels were measured after maternal injection of LIF at different doses (0, 1, 5, and 25 μg/kg BW; *n* = 4 or 6) with each point standardized to control (LIF 0 μg/kg BW). All data are expressed as mean ± SEM. *P < 0.05, ***P < 0.001 vs. control (Student’s *t*-test).

Next, we examined effects of LIF on CRH peptide level and *Crh* expression in mouse placenta *in vivo* (Figure 2). At 3 h after intraperitoneal injection of recombinant LIF into dams on 13.5 dpc, the CRH peptide level in mouse placenta was elevated (Figures 2A,B). Furthermore, placental *Crh* mRNA was upregulated by maternally injected LIF in a dose-dependent manner (Figure 2C).

### *Crh* Expression is Induced Prior to *Pomc* Expression by LIF in mTSCs

**FIGURE 3:**
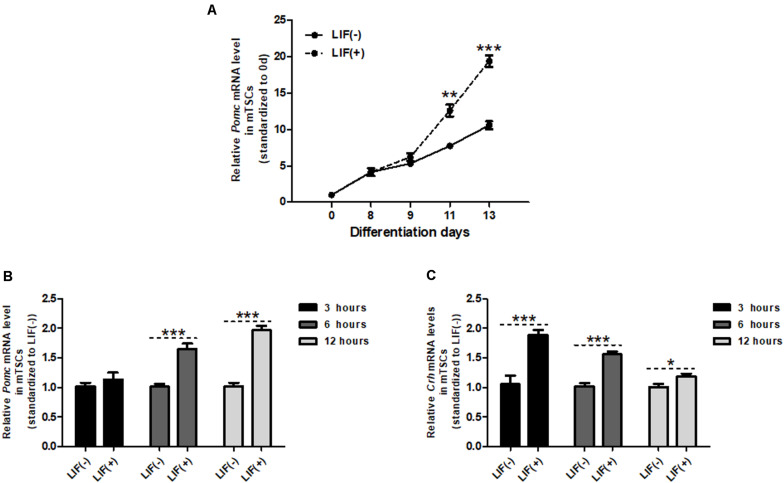
*Crh* expression is induced prior to *Pomc* expression by LIF in mTSCs. **(A)** Day 8 mTSCs were incubated with (+) or without (–) LIF (10 ng/mL). Quantitative RT-PCR analysis of *Pomc* expression during mTSC differentiation with (+) or without (–) LIF treatment (*n* = 5), with each point standardized to day 0. Each point was compared with LIF (–) mTSCs in the same group. Data are expressed as mean ± SEM. **P < 0.01, ***P < 0.001 vs. the LIF (*negative symbol*) group (Student’s *t*-test). **(B)** Real-time RT-PCR was used to measure *Pomc* mRNA expression level in mTSCs of LIF-treated group, with (+) or without (–) LIF for 3, 6, and 12 h (*n* = 5 or 6). Each point was compared with LIF (–) mTSCs in the same group. Data are expressed as mean ± SEM. ***P < 0.001 vs. LIF (*negative symbol*) mTSCs in the same group (Student’s *t*-test). **(C)** Real-time RT-PCR was used to measure *Crh* mRNA expression level in mTSCs, with (+) and without (–) LIF for 3, 6, and 12 h (*n* = 6 or 7). Each point was compared with LIF (–) mTSCs in the same group. Data are expressed as mean ± SEM. *P < 0.05, **P < 0.01, ***P < 0.001 vs. LIF (*negative symbol*) mTSCs in the same group (Student’s *t*-test).

Similar to *Crh* expression, *Pomc* mRNA levels in mTSCs gradually increased throughout differentiation and significantly increased after day 8 compared with day 0 (Figure 3A). We showed in previous studies that mTSCs differentiate into trophoblasts on day 8 in culture. Furthermore, after LIF treatment, *Pomc* mRNA levels increased significantly compared with that found in the control group without LIF treatment at day 11 and day 13. On day 11, *Crh* mRNA was upregulated 3 h after LIF treatment (Figure 3B), whereas *Pomc* mRNA was upregulated 6 h after treatment (Figure 3C). This finding supports the hypothesis that CRH is involved in LIF-induced upregulation of *Pomc* expression.

### ACTH Secretion Is Induced Directly by LIF, But *Pomc* Expression is Indirect via CRH/CRHR

**FIGURE 4:**
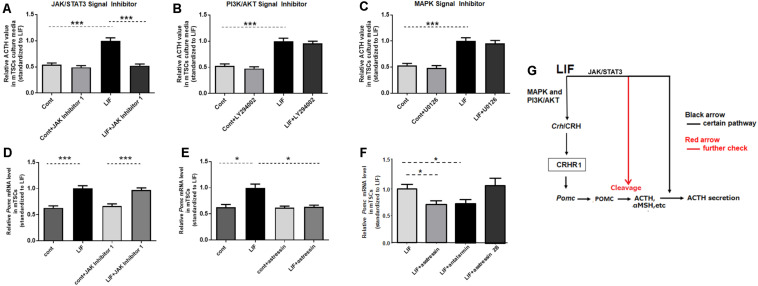
ACTH secretion is directly induced by LIF, but *Pomc* expression is indirectly induced via CRH/CRHR. Day 11 mTSCs cultured with LIF for 3 days were further incubated for 15 min or 6 h, with (+) or without (–) LIF, in the absence or presence of JAK Inhibitor 1, LY294002, U0126, or asstressin. **(A–C)** Relative ACTH concentrations in culture media in the absence or presence of JAK Inhibitor 1, LY294002, and U0126 (*n* = 5–8), with each point standardized to LIF (*positive symbol*) in the same group. Data are expressed as mean ± SEM. **(A)** Relative ACTH concentrations in the culture media in the absence or presence of JAK inhibitor 1. ***P < 0.001 between the cont and LIF groups (Student’s *t*-test); ***P < 0.001 between the LIF and LIF + JAK Inhibitor 1 groups (Student’s *t*-test). **(B)** Relative ACTH concentrations in the culture media in the absence or presence of LY294002. ***P < 0.001 between the cont and LIF groups (Student’s *t*-test). **(C)** Relative ACTH concentrations in the culture media in the absence or presence of U0126; ***P < 0.001 between the cont and LIF groups (Student’s *t*-test). **(D)** Real-time RT-PCR was used to measure *Pomc* mRNA expression levels in mTSCs, with (*positive symbol*) and without (*negative symbol*) LIF for 6 h in the absence or presence of JAK Inhibitor 1 (*n* = 6), with each point standardized to LIF in the same group. Data are expressed as mean ± SEM. ***P < 0.001 between the cont and LIF groups or between the cont + JAK Inhibitor 1 and LIF + JAK Inhibitor 1 groups (Tukey’s test). **(E)** Real-time RT-PCR was used to measure *Pomc* mRNA expression levels in mTSCs, with (*positive symbol*) and without (*negative symbol*) LIF for 6 h in the absence or presence of asstressin (*n* = 6), with each point standardized to LIF in the same group. Data are expressed as mean ± SEM. *P < 0.05 between the cont and LIF groups or between the LIF and LIF+ asstressin groups (Tukey’s test). **(F)** Real-time RT-PCR was used to measure *Pomc* mRNA expression levels in mTSCs, with (*positive symbol*) LIF for 6 h in the absence or presence of asstressin, antalarmin, and asstressin 2B (*n* = 6) with each point standardized to LIF in the same group. Data are expressed as mean ± SEM. *P < 0.05 between the LIF and LIF+ antalarmin groups or between the LIF and LIF+ asstressin groups (Student’s *t*-test). **(G)** Schematic diagram of the effect of LIF on *Pomc* expression and ACTH secretion in mouse trophoblasts.

To investigate the mechanism by which LIF promotes *Pomc* expression and ACTH secretion in mouse placenta, the downstream pathways involved in LIF activation in mTSCs were investigated *in vitro*. First, to identify the signal pathway for LIF-induced secretion of ACTH, the effects of JAK Inhibitor 1, LY294002, and U0126, which are specific inhibitors of JAK/STAT3, PI3K, and MAPK, respectively, were analyzed *in vitro*. We found that JAK Inhibitor 1, but not LY294002 and U0126, successfully inhibited LIF-induced ACTH secretion in mTSCs medium on day 11 (Figures 4A–C). This finding suggests that LIF directly induces ACTH secretion by the JAK/STAT3 pathway. Next, we investigated *Pomc* expression. Asstressin is a CRH antagonist blocking both CRHR1 and 2 that reduces ACTH synthesis in the pituitary. As shown in Figures 4D,E, asstressin completely blocked LIF-induced *Pomc* mRNA expression, whereas administration of JAK Inhibitor 1 had no effect on LIF-induced expression of *Pomc*. Next, this study determined whether the signaling pathway that induces *Pomc* expression in trophoblasts is CRHR1 or CRHR2 with specific inhibitors of CRHR1 or CRHR2 *in vitro*. Figure 4F shows that antalarmin, which is a specific CRHR1 inhibitor, successfully blocked LIF-CRH/CRHR-induced *Pomc* expression, whereas asstressin 2B did not inhibit *Pomc* expression. The dose–response effects of asstressin 2B on *Pomc* expression were examined but confirmed to have no effect (Supplementary Figure 1). These findings indicate that the effect of LIF on *Pomc* expression is indirect via the CRH/CRHR1 pathway.

## Discussion

In this study, we discovered that CRH is expressed in mouse placenta *in vivo*, and that CRH and LIF protein levels peak at 13.5 dpc. We also determined that LIF indirectly increases *Pomc* expression in mouse trophoblasts through the CRH/CRHR pathway *in vitro*, whereas LIF-induced ACTH is directly dependent on the JAK/STAT3 pathway in mouse trophoblast cells *in vitro*.

CRH is a releasing hormone mainly produced in the hypothalamus. In addition to the central nervous system, the placenta expresses CRH (Grino et al., 1987). In human placenta, CRH biosynthesis and secretion from trophoblasts gradually increase until the end of pregnancy (Smith et al., 2012). Previous studies have shown that only anthropoid primate species express placental CRH (Bowman et al., 2001); however, because of advancements in instrumentation, several researchers have since quantified murine placental *Crh* mRNA and protein (Wang et al., 2016, 2020; Zhou et al., 2016). Consistent with these findings, we also found CRH in mouse placenta. In this study, CRH immunostaining showed that the syncytiotrophoblasts at 13.5 dpc showed strong immunopositivity for CRH peptide, which gradually decreased at day 17.5 (Figures 1B,C). The temporal and spatial LIFR expression patterns were consistent with those of the CRH peptide. Interestingly, and different from human placental CRH, the level of *Crh* mRNA is highest at 13.5 dpc and gradually decreases as the pregnancy progresses, particularly in late pregnancy. This finding may also explain why many studies have suggested that CRH is not expressed in murine placenta.

LIF is the most pleiotropic member of the IL-6 family of cytokines. After binding to its receptor gp130 (also known as Il6st), LIF initiates a cascade of tyrosine phosphorylation that stimulates three distinct signaling pathways, the JAK/STAT3, MAPK, and PI3K/AKT pathways (Fahmi et al., 2013), among which the MAPK and PI3K/AKT pathways are involved in LIF-induced upregulation of CRH in mTSCs (Wang et al., 2020). JAK/STAT3 is the most commonly stimulated pathway by LIF; this pathway is involved in LIF-induced *Pomc* expression and ACTH secretion in murine trophoblasts and cells from a corticotroph cell line (Simamura et al., 2010; Tsukada et al., 2015). In this study, we further proved that LIF induces upregulation of CRH in mouse placenta *in vivo.* LIF-induced *Pomc* expression and ACTH secretion from mouse placenta was confirmed before (Simamura et al., 2010; Tsukada et al., 2015); however, in our study, we determined whether CRH, which is the most common agonist of ACTH in brain, plays a role in *Pomc* expression and ACTH secretion caused by LIF. Interestingly, we found that *Pomc* expression induced by LIF was normalized by inhibition of CRHR1. However, this upregulation of *Pomc* expression didn’t change despite suppression of the JAK/STAT3 pathway, which suggests that the JAK/STAT3 signaling pathway is not involved in LIF-induced upregulation of *Pomc*. In other words, LIF indirectly induces *Pomc* expression via the CRH/CRHR1 pathway, but not directly by the JAK/STAT3 pathway (Figure 4G).

Corticotropin-releasing hormone is also expressed in the decidua in addition to trophoblasts based on the current study. Previous studies have shown that CRH signaling via CRHR1 in the trophoblast and decidua contributes to the promotion of implantation and maintenance of early pregnancy in humans by regulating T cell activation (Makrigiannakis et al., 2001, 2003). However, whether this scheme in humans could be directly extrapolated to rodent pregnancy is still unclear, and detailed pregnant stage-specific analysis *in vivo* should be addressed. The present assay used mTSCs, a trophoblast-lineage not containing decidual cells. Also, the current study *in vivo* only focused on the CRH/CRHR-POMC pathway in trophoblasts. Therefore, the co-culture system with mTSCs and decidual cell derivatives may be effective in the future to address the issues on decidual cells.

POMC is the precursor protein of ACTH, which is not stable and is proteolytically cleaved to a variety of neuroendocrine peptides, including ACTH. After 15 min of LIF supplementation, we found a demonstrable increase of ACTH in mTSC culture medium. Furthermore, this response from LIF to ACTH secretion is rapid. Based on our research, the synthesis of new *Pomc* takes at least 6 hours after LIF treatment. Therefore, the effect of LIF on ACTH is likely a direct effect on the secretion of ACTH already stored in the cell and not newly synthesized. Our results reveal that this rapid secretion of ACTH caused by LIF is dependent on the JAK/STAT3 pathway, but not the PI3K/AKT and MAPK pathways. Our present findings are consistent with those of previous studies of cells from the murine AT-20 corticotroph cell line (Ray et al., 1996; Chesnokova and Melmed, 2002). Therefore, LIF directly induces ACTH secretion from mouse trophoblast (Figure 4G), but the question regarding the effect of LIF-induced CRH on ACTH secretion remains, and is a limitation of the current study as we did not measure ACTH concentration in culture medium for longer than 3 h after LIF treatment. Thus, greater elucidation of the LIF/CRH/ACTH pathway warrants further research.

Previous studies revealed temporary increases in LIF levels in amniotic fluid and maternal serum of humans and rodents during pregnancy (Hatta et al., 2006; Simamura et al., 2010). In the present study, an artificial increase in LIF levels triggers increased CRH expression and ACTH secretion from mouse trophoblasts. Based on our findings from previous studies, we hypothesized that these peaks of LIF are involved in a cascade of physiological processes. Maternal LIF binds to gp130 and the LIFR on mouse trophoblast, resulting in the activation of three downstream signals namely, JAK/STAT3, PI3K/AKT, and MAPK. As a result, *CRH* expression is upregulated via the PI3K/AKT and MAPK signals, and CRH-induced *Pomc* expression in trophoblast is involved in ACTH secretion through an autocrine effect. In addition, LIF directly induces ACTH secretion by the JAK/STAT3 pathway. The newly produced CRH and ACTH from trophoblast accounts for different functions in fetal and maternal circulation, having particularly important roles in fetal brain and other organs.

**FIGURE 5:**
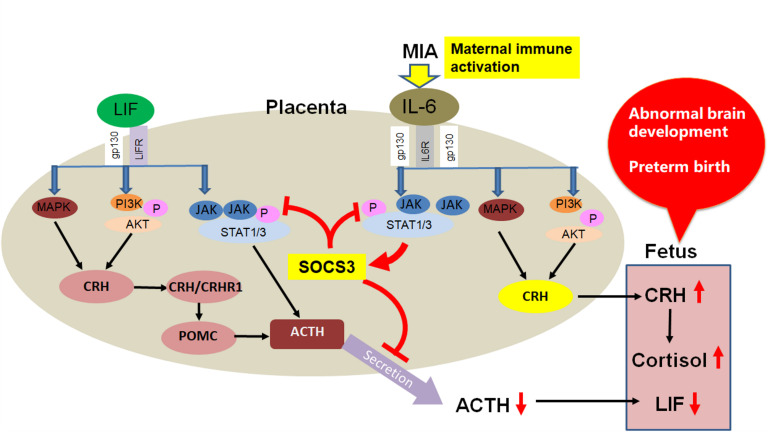
Proposed IL-6 family cytokine-based mechanism of fetal neurodevelopmental disorders induced by MIA.

Based on research findings of the LIF/gp130 downstream pathway, we propose there are IL-6 family cytokine-based mechanisms of fetal neurodevelopmental disorders induced by maternal immune activation (MIA) (Figure 5). In contrast to LIF, IL-6 is induced under a pathological state. After MIA induction by infection, IL-6 in maternal serum is elevated, and placental SOCS3 and CRH are upregulated in response to the increased IL-6 (Wu et al., 2017; Tsukada et al., 2018). As SOCS3 negatively regulates the JAK/STAT3 pathway, placental ACTH secretion is inhibited leading to decreased fetal LIF. Further, because IL-6 induced upregulation of CRH is independent of the JAK/STAT3 pathway, SOCS3 is unable to inhibit CRH upregulation induced by maternal IL-6. Continuously elevated CRH can enter the fetus through the placenta, leading to elevated cortisol. This aberrant and persistent upregulation of CRH and downregulation of LIF in the mother and fetus leads to adverse brain development and preterm birth. Admittedly, our hypothesis needs further study; however, it may provide the basis for new treatments of premature delivery and fetal brain developmental abnormalities caused by infection during pregnancy.

## Conclusion

We discovered that LIF induces CRH in mouse trophoblasts and placenta, and that this upregulation of CRH involves *Pomc* expression induced by LIF. In other words, LIF indirectly induces *Pomc* expression via the CRH/CRHR1 pathway, and directly induces ACTH secretion from mouse trophoblasts. This mechanism may contribute to fetal brain development under a physiological state during pregnancy.

## Data Availability Statement

The raw data supporting the conclusions of this article will be made available by the authors, without undue reservation.

## Ethics Statement

The animal study was reviewed and approved by the Ethics Committee on Animal Experiments of the Kanazawa Medical University.

## Author Contributions

HW, TT, HShi, HS-H, DS, and HSho performed the experiments. TH and HW designed research, analyzed data, and edited the manuscript for intellectual content. HM, MT, and DS performed the Supplementary experiments. All authors read and approved the final manuscript.

## Conflict of Interest

The authors declare that the research was conducted in the absence of any commercial or financial relationships that could be construed as a potential conflict of interest.

## Supplementary Material

Supplementary Figure 1 Analysis of dose-dependent effects of asstressin 2B on *Pomc* induction via LIF signaling. Real-time RT-PCR was used to measure *Pomc* mRNA expression levels in mTSCs with different doses of asstressin 2B (*n* = 6), with each point standardized to LIF in the same group. Data are expressed as mean ± SEM. No remarkable inhibitory effects of asstressin 2B, a specific CRHR2 inhibitor, were observed on the induction of *Pomc* expression under LIF stimulation.

## Abbreviations

ACTH: adrenocorticotropic hormone
CRH: corticotropin-releasing hormone
CRHR: corticotropin-releasing hormone receptor
BW: body weight
gp130: glycoprotein 130
IL-6: interleukin-6
JAK/STAT3: Janus kinase/signal transducer and activator of transcription 3
LIF: leukemia inhibitory factor
MAPK: mitogen-activated protein kinase
mTSCs: mouse trophoblast stem cells
MIA: maternal immune activation
POMC: proopiomelanocortin
PI3K/AKT: phosphatidylinositol 3 kinase/protein kinase B
SOCS3: suppressor of cytokine signaling 3.
