# Supplementary figures and images for: Leukemia Inhibitory Factor Induces Proopiomelanocortin via CRH/CRHR Pathway in Mouse Trophoblast
Source: Front Cell Dev Biol. 2021 Jul 19;9:618947. doi: 10.3389/fcell.2021.618947 (PMC8326836; doi:10.3389/fcell.2021.618947)

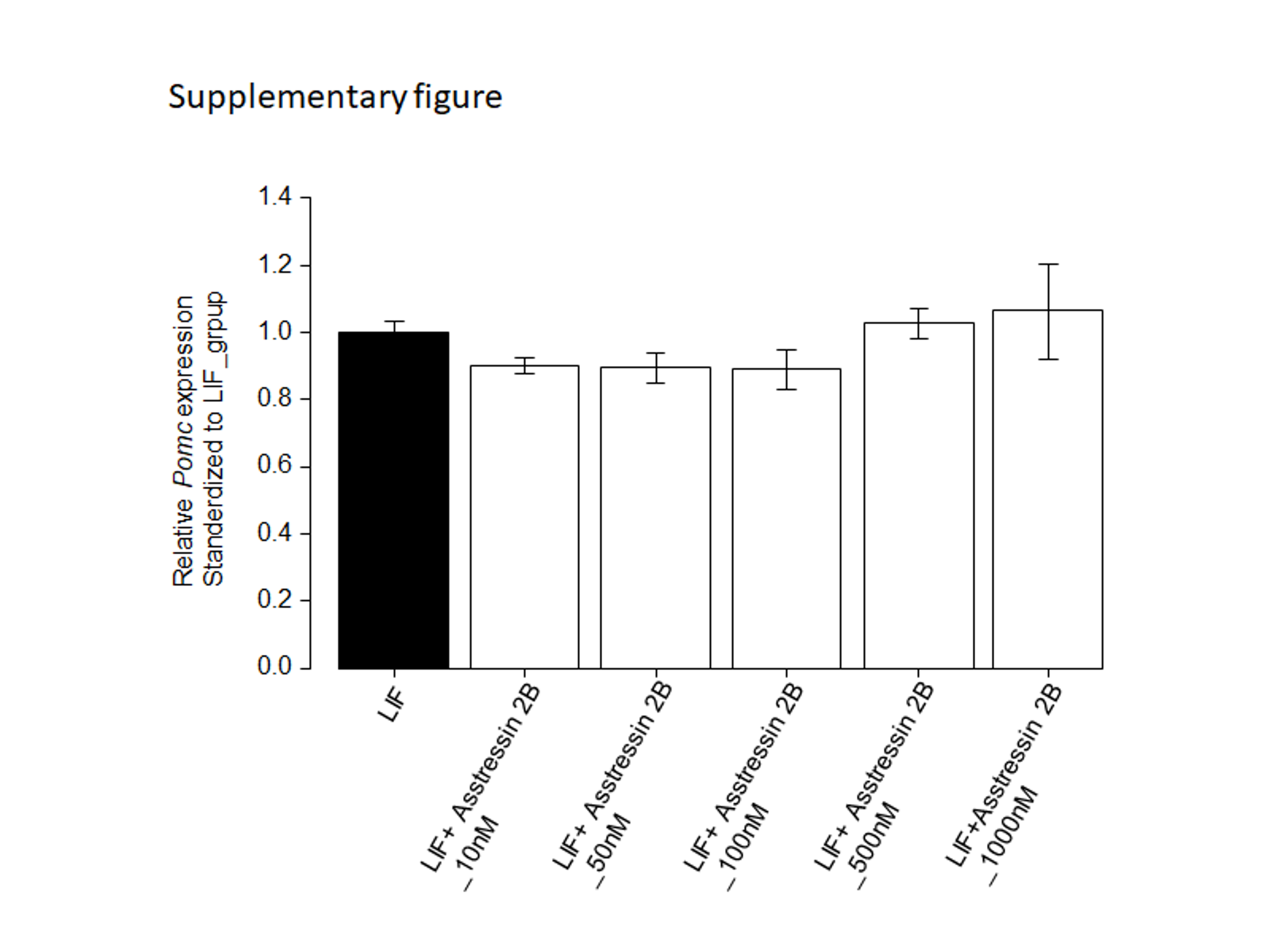

Supplement: Supplementary Figure 1 — Analysis of dose-dependent effects of asstressin 2B on Pomc induction via LIF signaling. Real-time RT-PCR was used to measure Pomc mRNA expression levels in mTSCs with different doses of asstressin 2B (n = 6), with each point standardized to LIF in the same group. Data are expressed as mean ± SEM. No remarkable inhibitory effects of asstressin 2B, a specific CRHR2 inhibitor, were observed on the induction of Pomc expression under LIF stimulation. [file Image_1.TIF]
